# Scapular Osteoid Osteoma: An Extremely Rare Case with an Atypical Clinical Presentation

**DOI:** 10.3390/diagnostics16213509

**Published:** 2026-10-27

**Authors:** Kouhei Mitsui, Jiro Ichikawa, Kojiro Onohara, Tomonori Kawasaki, Satoshi Kanno, Masanori Wako, Rikito Tatsuno, Taro Fujimaki, Tetsuhiro Hagino, Hirotaka Haro

**Affiliations:** 1 Department of Orthopaedic Surgery, Interdisciplinary Graduate School of Medicine, University of Yamanashi, Chuo, Yamanashi 4093898, Japan; koumitsui@yamanashi.ac.jp (K.M.); wako@yamanashi.ac.jp (M.W.); rtatsuno@yamanashi.ac.jp (R.T.); tfujimaki@yamanashi.ac.jp (T.F.); t-hagino@yamanashi.ac.jp (T.H.); haro@yamanashi.ac.jp (H.H.); 2 Department of Diagnostic Radiology, Interdisciplinary Graduate School of Medicine, University of Yamanashi, Chuo, Yamanashi 4093898, Japan; konohara@yamanashi.ac.jp; 3 Department of Pathology, Saitama Medical University International Medical Center, Hidaka, Saitama 350-1298, Japan; tomo.kawasaki.14@gmail.com (T.K.); kanno820@saitama-med.ac.jp (S.K.)

**Keywords:** osteoid osteoma, scapula, histopathology, imaging, differential diagnosis

## Abstract

Osteoid osteoma (OO) is a benign osteoblastic tumor that rarely occurs in the scapula and may present with atypical clinical and imaging findings. A 25-year-old woman presented with a 2-month history of right shoulder pain without nocturnal pain. Radiographs revealed a well-defined osteolytic lesion with internal calcification and surrounding osteosclerosis in the scapular body. Computed tomography (CT) demonstrated a 15-mm lesion containing an 11-mm coarse calcification, whereas magnetic resonance imaging showed surrounding bone marrow edema. The differential diagnosis included OO and osteomyelitis. Surgery was performed for pain relief and definitive diagnosis. Histopathological examination revealed immature woven bone trabeculae within a fibrovascular stroma, with prominent osteoblastic rimming and minimal cytologic atypia, confirming the diagnosis of OO. The patient remained asymptomatic at the 1-year follow-up, and radiographs showed no evidence of local recurrence. OO of the scapular body is extremely rare and may present without the characteristic nocturnal pain. CT is particularly useful for identifying the nidus, whereas histopathological examination remains important for definitive diagnosis.

**Figure 1:**
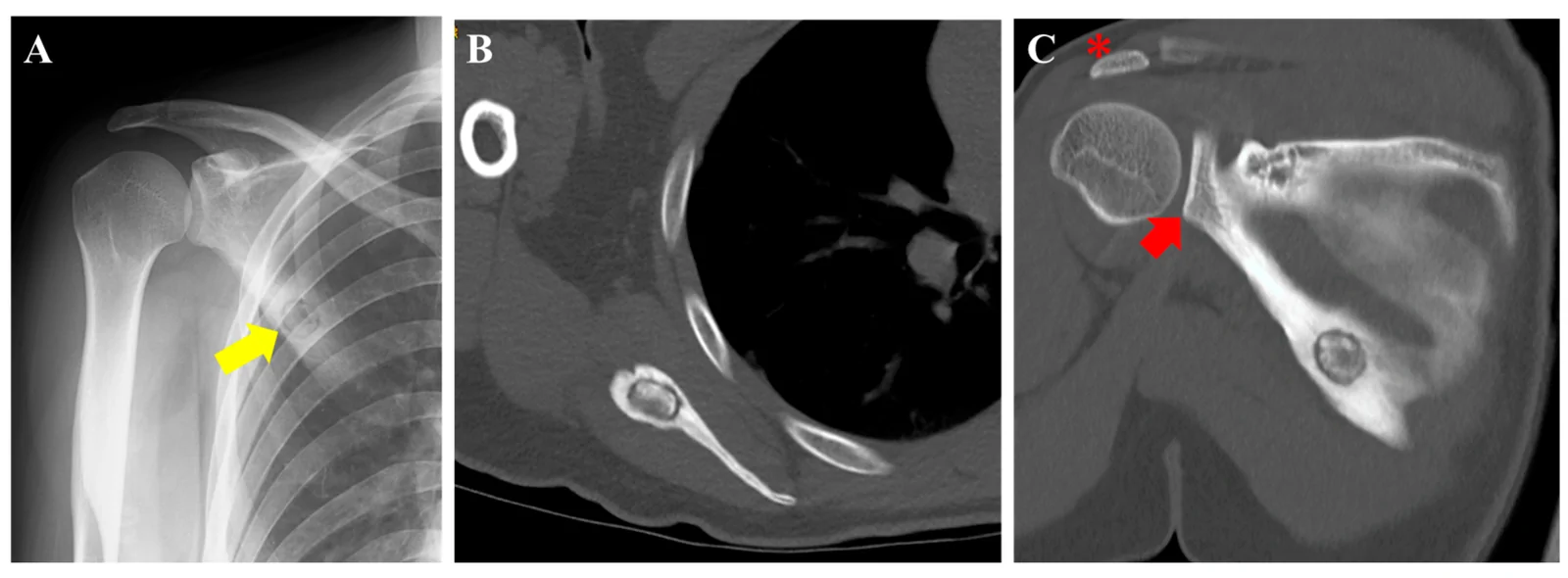
A 25-year-old woman presented to our hospital with a 2-month history of right shoulder pain. She initially visited a local orthopedic clinic, where radiographs revealed a suspected bone tumor, prompting referral to our hospital for further evaluation. Physical examination showed no erythema or swelling. Although no tenderness was noted, she reported spontaneous pain extending from the right shoulder to the scapula. This pain, which occurred during the daytime and was rarely present at night, was adequately controlled with nonsteroidal anti-inflammatory drugs and did not interfere with the patient’s daily activities or work. Shoulder range of motion was unrestricted. Laboratory tests, including white blood cell count, C-reactive protein, and alkaline phosphatase levels, were within normal limits. She had no relevant medical, surgical, or trauma history. On plain radiography ((**A**), yellow arrow), an osteolytic lesion with internal calcification was observed at the lateral margin of the right scapular body, accompanied by surrounding osteosclerosis. Computed tomography ((**B**,**C**), red arrow; glenoid, red asterisk; acromion) demonstrated a well-defined, 15-mm osteolytic lesion at the lateral margin of the right scapula. The lesion contained an 11-mm coarse calcification and extensive surrounding sclerosis of the adjacent bone.

**Figure 2:**
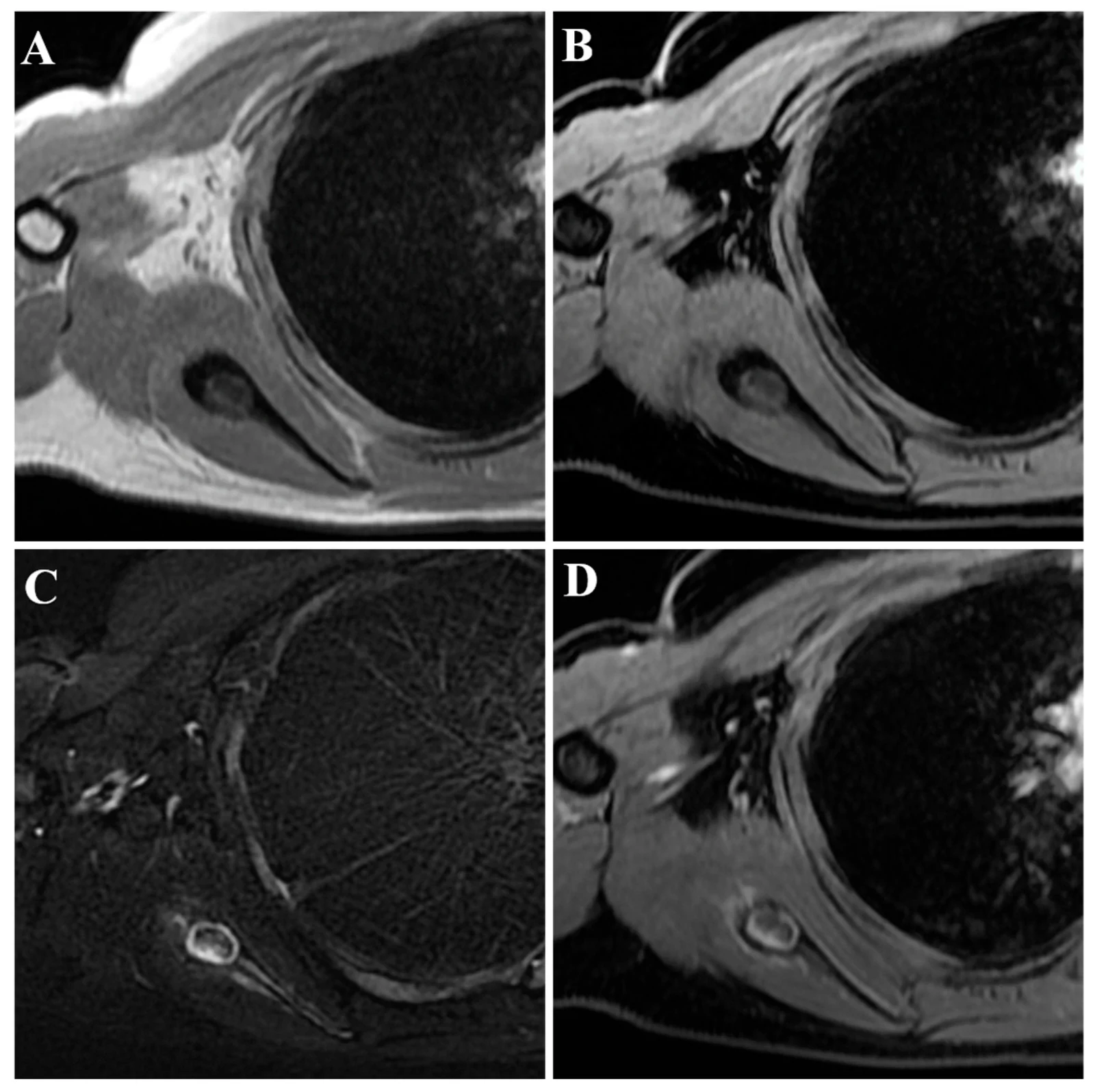
On magnetic resonance imaging, the internal calcification showed low signal intensity relative to muscle on T1-weighted (**A**), fat-suppressed T1-weighted (**B**), and short-tau inversion recovery (STIR) (**C**) images. The peripheral portion of the lesion showed signal intensity similar to that of muscle on T1-weighted and fat-suppressed T1-weighted images and high signal intensity on STIR images, with contrast enhancement (**D**). Edematous changes were also observed in the surrounding bone marrow. Based on these imaging findings, the differential diagnosis included bone tumors, particularly osteoid osteoma (OO), and inflammatory conditions such as osteomyelitis.

**Figure 3:**
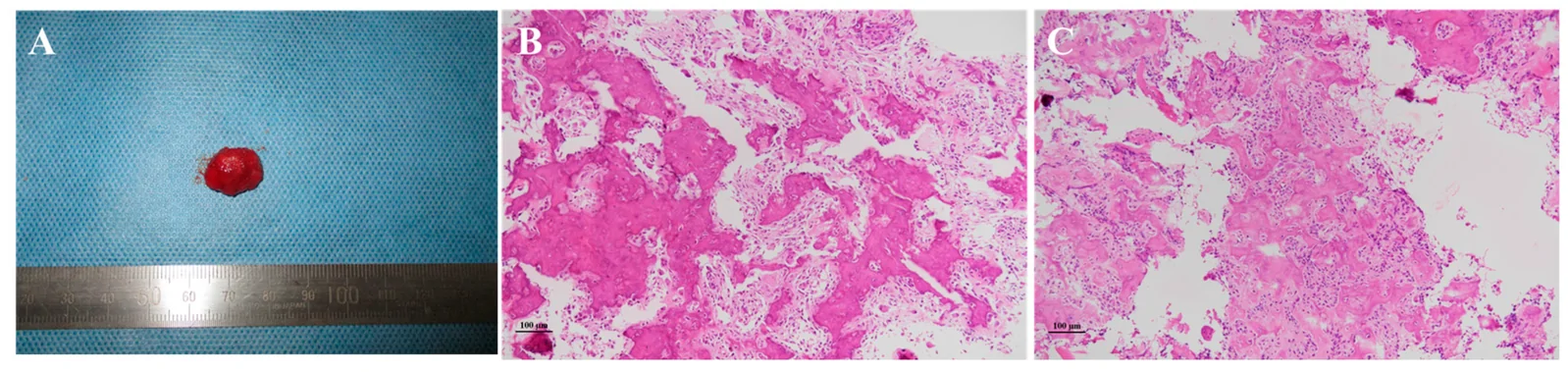
Surgery was performed for pain relief and definitive diagnosis. Because preoperative biopsy under local anesthesia was deemed difficult, we planned to perform this procedure under general anesthesia, with intraoperative frozen-section analysis planned to establish the diagnosis. Under general anesthesia, the lesion was approached posteriorly by dividing the overlying muscles. The location of the lesion was confirmed intraoperatively using an O-arm imaging system, and the nidus was carefully localized before en bloc resection. Intraoperative frozen-section analysis revealed no malignant features, and the lesion was therefore subsequently resected en bloc. Gross examination showed a firm, elastic mass measuring 15 × 12 × 10 mm (**A**). Histologically, the lesion comprised irregularly arranged and interanastomosing trabeculae of immature woven bone within a fibrovascular stroma containing numerous small capillaries. The bony trabeculae showed prominent osteoblastic rimming, with plump osteoblasts lining the trabeculae, without any significant cytologic atypia ((**B**), ×100; (**C**), ×100). These histological findings were all consistent with osteoid osteoma.

**Figure 4:**
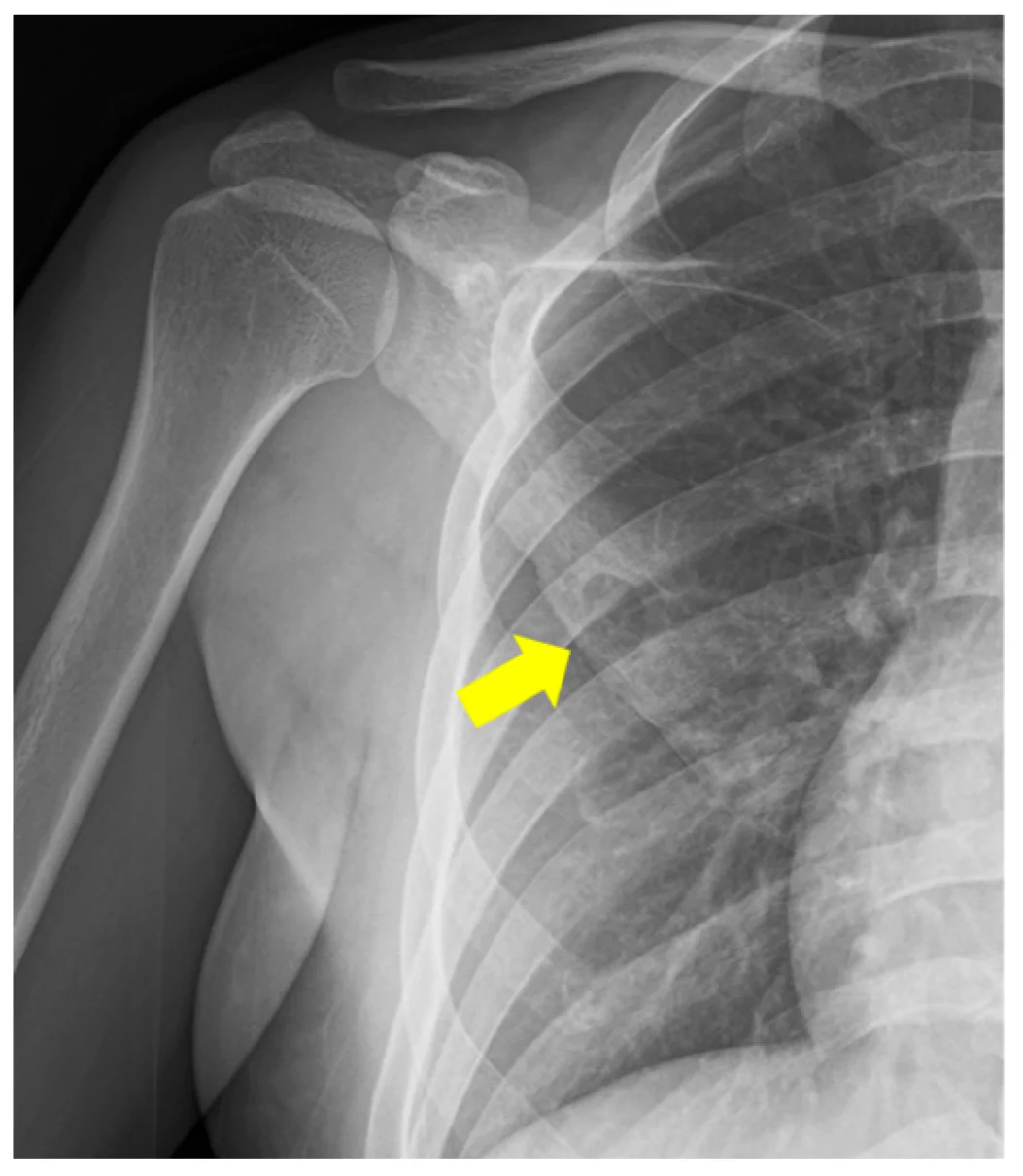
The patient used NSAIDs for approximately 4 weeks after surgery, but did not require them thereafter. The patient remained asymptomatic at the 1-year follow-up, and follow-up radiographs showed no evidence of any local recurrence (yellow arrow). Osteoid osteoma (OO) is a benign osteoblastic tumor that accounts for approximately 3% of all primary bone tumors. It most commonly occurs in the diaphysis or metaphysis of the long bones of the lower extremities in children and young adults. OO has a male predominance, and the lesion is typically smaller than 2 cm. Nocturnal pain is a characteristic symptom of OO [1,2]. The diagnostic significance of the present case lies not simply in the rarity of the scapular location, but also in the combination of an unusual anatomical site with an atypical clinical presentation. The absence of any nocturnal pain and the relatively prominent calcified component may further reduce the clinical suspicion of OO, despite the presence of a characteristic nidus on CT. Scapular involvement in OO is uncommon, representing approximately 1% of all reported cases. The incidence of OO arising in the scapular body remains unclear, as only a limited number of cases have been reported in the literature [3]. To the best of our knowledge, most previously reported cases of scapular OO have involved the glenoid [4], coracoid process [5], or acromion [6], whereas involvement of the scapular body is exceedingly rare. Scapular bone tumors are themselves uncommon. Priemel et al. reported a mean patient age of 38 years and a slight male predominance, with males and females accounting for 58% and 42% of cases, respectively [7]. Benign, intermediate, and malignant tumors accounted for 31%, 15%, and 54% of cases, respectively [7]. The most common histological types were osteochondroma, chondrosarcoma, metastatic bone tumor, and Ewing sarcoma, in descending order of frequency. Cartilaginous tumors accounted for up to 47% of all scapular bone tumors. Although scapular bone tumors account for only approximately 1.6% of all bone tumors, careful evaluation is warranted because malignant tumors constitute a relatively high proportion of these lesions [7]. The characteristic imaging feature of OO is the presence of a nidus. In the present case, MRI demonstrated extensive bone marrow edema and enhancement around the lesion, which may overlap with inflammatory or malignant conditions. In contrast, CT clearly demonstrated a well-defined nidus with central calcification and surrounding sclerosis, providing more specific clues to diagnose OO. CT is considered superior to MRI for detecting the nidus, and careful evaluation of CT findings may be particularly important when OO occurs at an atypical skeletal site [8]. The differential diagnosis of OO based on imaging findings is broad and includes both neoplastic and inflammatory conditions [9,10]. Neoplastic lesions that can mimic OO include chondroblastoma, glomus tumors, fibrous dysplasia, and metastatic bone tumors, while possible non-neoplastic conditions include stress fractures and infectious lesions, such as Brodie abscesses or noninfectious inflammatory lesions [9,10]. Bone marrow edema, which is frequently observed in OO, may mimic the imaging appearance of inflammatory disease or malignancy on MRI. Therefore, CT and MRI should be considered complementary modalities when establishing the diagnosis [9]. Intra-articular OO may be particularly difficult to diagnose because it can lack cortical thickening and periosteal reaction; therefore, lesions in this location require particular attention [8]. Because the imaging findings of OO can overlap with those of other neoplastic and inflammatory conditions, histopathological examination is essential for definitive diagnosis. Histologically, the central nidus consists of immature woven bone with focal osteoblastic rimming. The fibrous tissue between the bony trabeculae contains scattered osteoclasts and small- to medium-sized vascular channels. A rim of mature, compact bone is formed around the lesion, surrounding the nidus [1]. These histological features may overlap with those of osteoblastomas, making histological distinction between the two entities challenging [11]. Osteoblastoma generally occurs in lesions measuring 2 cm or larger, has a predilection for the spine, and is less frequently associated with pain and less responsive to NSAIDs. On imaging, osteoblastomas generally present as less prominent peripheral reactive sclerosis than osteoid osteomas. However, lesion size alone is insufficient to distinguish osteoblastomas from OOs, particularly lesions measuring 1–2 cm. Accordingly, diagnosis should be based on a comprehensive assessment of the anatomical site, imaging characteristics, and clinical features [11,12]. In the present case, the small lesion size (15 mm), well-defined nidus with central calcification, prominent surrounding sclerosis on CT, and clinical presentation all favored osteoid osteoma over osteoblastoma. Treatment options for OO include conservative management, surgical excision, and minimally invasive percutaneous procedures. Historically, open surgery, including en bloc resection and curettage, was the standard treatment for symptomatic lesions [1]. Although these procedures can achieve complete nidus removal and provide tissue for histopathological examination, they may require extensive dissection and are associated with potential complications, including postoperative fracture and the need for internal fixation [1]. CT-guided percutaneous excision or curettage has also been used as a minimally invasive alternative in selected cases. More recently, CT-guided percutaneous ablation techniques, including radiofrequency ablation, cryoablation, and microwave ablation, have become widely used because of their minimally invasive nature [1]. Percutaneous ablation has been reported to achieve treatment success rates exceeding 90%, which are broadly comparable to the reported success rates of 83–100% for CT-guided percutaneous excision [1]. However, potential complications include skin burns, hematoma, and nerve injury. Furthermore, ablation may compromise subsequent histopathological evaluation; therefore, biopsy should be considered before ablation when a definitive diagnosis is uncertain [1]. In the present case, en bloc resection was selected as the lesion was extraarticular, located in a non-weight-bearing portion of the scapula, and relatively easy to access surgically, allowing for a definitive histopathological diagnosis and complete removal of the nidus. The present case highlights an important diagnostic consideration for OO of the scapular body. Although a characteristic nidus was identified on CT, the absence of any nocturnal pain and prominent bone marrow edema on MRI resulted in atypical clinical and imaging findings. Accordingly, careful correlation of clinical findings with CT, MRI, and histopathological findings is important when evaluating small osteolytic lesions of the scapula. In particular, the recognition of a well-defined nidus with internal calcification on CT may facilitate the diagnosis of OO, even in the absence of characteristic nocturnal pain.

## Data Availability

The original contributions of this study are included in the article. Further inquiries can be directed to the corresponding author(s).

## Abbreviations

The following abbreviations are used in this manuscript:

OO	Osteoid osteoma
CT	Computed tomography
MRI	Magnetic resonance imaging
STIR	Short-tau inversion recovery

## References

[1] Tepelenis K., Skandalakis G.P., Papathanakos G., Kefala M.A., Kitsouli A., Barbouti A., Tepelenis N., Varvarousis D., Vlachos K., Kanavaros P. (2021). Osteoid osteoma: An updated review of epidemiology, pathogenesis, clinical presentation, radiological features, and treatment option. In Vivo.

[2] Napora J., Wałejko S., Mazurek T. (2023). Osteoid osteoma, a diagnostic problem: A series of atypical and mimicking presentations and review of the recent literature. J. Clin. Med..

[3] Goyal S., Said H.G. (2015). Osteoid osteoma of the coracoid: A case report of arthroscopic excision and review of literature. SICOT J..

[4] Amin Z.H.F., Al-Rasheedi R.S., Sairafi M.H., Alrashidi Y. (2020). Osteoid osteoma of scapular glenoid: A case report. Int. J. Surg. Case Rep..

[5] Ueyama H., Ito Y., Manaka T., Matsumoto I., Ichikawa K., Nakamura H. (2016). Arthroscopic treatment of an osteoid osteoma at the base of the coracoid process: A case report. J. Orthop. Sci..

[6] Degreef I., Verduyckt J., Debeer P., De Smet L. (2005). An unusual cause of shoulder pain: Osteoid osteoma of the acromion—A case report. J. Shoulder Elb. Surg..

[7] Priemel M.H., Erler J.M.E., Zustin J., Luebke A.M., Stiel N., Spiro A.S. (2019). Histological, epidemiological and anatomical analysis of 193 bone tumours of the scapula. J. Bone Oncol..

[8] Bedoya M.A., Iwasaka-Neder J., Tsai A., Bixby S.D. (2024). Intra-articular osteoid osteomas: Imaging manifestations and mimics. RadioGraphics.

[9] Gillet R., Boubaker F., Gauchotte G., Clara M., Talabard M.P., Teixeira P.A.G., Douis N., Louis M., Blum A. (2026). Diagnostic and interventional imaging of osteoid osteoma: State-of-the-art. Diagn. Interv. Imaging.

[10] Carneiro B.C., Da Cruz I.A.N., Ormond Filho A.G., Silva I.P., Guimarães J.B., Silva F.D., Nico M.A.C., Stump X.M.G.R.G. (2021). Osteoid osteoma: The great mimicker. Insights Imaging.

[11] Amary F., Bredella M.A., Horvai A.E., Mahar A.M. (2020). Osteoblastoma. WHO Classification of Tumours.

[12] Yalcinkaya U., Doganavsargil B., Sezak M., Kececi B., Argin M., Basdemir G., Oztop F. (2014). Clinical and Morphological Characteristics of Osteoid Osteoma and Osteoblastoma: A Retrospective Single-Center Analysis of 204 Patients. Ann. Diagn. Pathol..

